# Factors Associated with the Extent of Coronary Artery Disease and the Attained Outcome of Percutaneous Coronary Intervention at Gesund Cardiac and Medical Center, Addis Ababa, Ethiopia

**DOI:** 10.4314/ejhs.v32i3.9

**Published:** 2022-05

**Authors:** Bekele Alemayehu Shashu, Ararso Baru

**Affiliations:** 1 Department of Internal Medicine, College of Health Science, Addis Ababa University, Addis Ababa, Ethiopia; 2 College of Medicine and Health Sciences, Arbaminch University, Arbaminch, Ethiopia

**Keywords:** coronary artery disease, percutaneous coronary intervention, Ethiopia

## Abstract

**Background:**

In Ethiopia, objective evidences showing pathologic features and clinical characteristics predicting the extent of coronary artery disease are scanty. The present study is aimed at assessing factors associated with the extent of coronary artery disease and the attained outcomes in patients undergoing percutaneous coronary intervention.

**Methods:**

A retrospective observational study of 197 patients that have undergone percutaneous coronary intervention was undertaken. Data were entered using Epi data version 4.2 and exported to Statistical Package for Social Science version 25 for analysis. Descriptive statistics such as frequencies and percentages were used to summarize the findings. Logistic regression was carried out to test the association between dependent and independent variables.

**Results:**

The mean age of the study participants was 58.6 with standard deviation (SD) of 11.5 and male to female ratio of 4.2. The majority, [110 (55.8%)], had ST segment elevation myocardial infarction. Nearly two-third of the study participants had documented heart failure. Dyslipidemia [AOR 4.2(95%; CI:1.29–14.00)] and left ventricular hypertrophy [AOR 4.1(95%; CI:1.38–12.40)] were associated with extent of coronary artery disease on adjusted analysis. In the large majority, 169 patients (85.8%), thrombolysis in myocardial infarction 3 flow grade was achieved.

**Conclusion:**

Dyslipidemia and left ventricular hypertrophy predicted multi-vessel coronary artery disease. There is a high frequency of post myocardial infarction heart failure, underscoring the need for centers of excellence and implementation of health education programs targeting the importance of primary prevention and timely revascularization. The success rate of percutasneous coronary intervention at Gesund Cardiac and Medical Center is praiseworthy.

## Introduction

Coronary artery disease (CAD), as a result of the atherosclerotic process in coronary arteries, is the leading cause of morbidity and mortality worldwide (1). Coronary atherosclerosis may be detected early in life, but it usually progresses from subclinical asymptomatic to clinically overt disease over several decades. The progressive obstruction of coronary arteries and the subsequent severe restriction of blood flow to the myocardium lead to the appearance of chronic ischemia and symptoms of chronic coronary syndrome (CCS). Rupture of a vulnerable atherosclerotic plaque along with the superimposition of thrombus is the major pathophysiological mechanism leading to acute coronary syndromes (ACS); unstable angina and acute myocardial infarction (AMI) (2, 3).

Acute myocardial infarction (AMI) is classified according to ST segment changes on electrocardiography (ECG) amongst the two main types: ST Segment Elevation Myocardial Infarction (STEMI) and non-ST Segment Elevation Myocardial Infarction (NSTEMI). STEMI is defined as new ST elevation at the J point ≥1 mm in at least 2 contiguous chest leads, other than V2-V3, or limb leads. STEMI is also defined as ≥2 mm ST elevation in men age 40 or above or > 2.5 mm in healthy men under 40 or ≥1.5 mm in women in leads V2-V3 (4). It accounts for about 38% of all myocardial infarction in the United States (5).

Although larger studies showing communitywide magnitude of the problem are lacking in Ethiopia, CAD is documented as a significant health problem in studies done among patients with the entire spectrum of cardiovascular diseases (CVD) in hospital-based practices (6,7). Objective evidences of CAD displaying the pathologic features and clinical characteristics predicting the extent of the disease are scanty (8,9). In the analysis of postmortem study results of victims of sudden death in Addis Ababa, Ethiopia, CAD accounted for 70% of cases (8). To our knowledge, our previous study is the only one that has recently tried to define the magnitude of the problem based on coronary angiographic findings. In this study, CAD was shown to manifest at an average age of 56 with a large majority of the patients presenting with ACS, most frequently STEMI (9). Considerable proportion of patients also had CCS presenting with typical angina and angina equivalents. In the same study, percutaneous coronary intervention (PCI) without surgical back up has been confirmed effective against a high frequency of STEMI on one hand and multivessel disease on the other, the two most troubling pathological conditions in the course of performance of PCI (9).

The present study was done to further substantiate the results on a larger group of patients that have undergone PCI with the accruing experience, better devices and refined adjuvant pharmacology through time. It attempts to define the clinical features and noninvasive investigation findings correlated with the extent of CAD. It also addresses the variables determining the outcome of PCI in Ethiopia, without discernable surgical back up.

## Methods

This single center retrospective observational study was conducted at Gesund Cardiac Medical and Center (GCMC), a private cardiac facility in Addis Ababa, Ethiopia, equipped with state-of-the-art catheterization laboratory (Cath-Lab). The center provides with various cardiac services to the people of Ethiopia, Eritrea, Somalia and Djibouti. The study was conducted through reviewing medical records of all patients with CAD who underwent PCI between June 2018 and February 2021 at GCMC. A data collection tool was developed to extract data, including demographic variables, past medical history, presenting complaints, medication at presentation, electrocardiography findings, echocardiography findings, clinical diagnosis, coronary angiography findings and complications of PCI. The data were collected by internists working in the center with good knowledge of cardiovascular medicine.

The data were checked for completeness, cleaned, coded, and entered into EPI data version 4.2 for validation. Statistical Package for Social Science (SPSS) version 25 was used for the analysis of the data. Descriptive analysis was performed to summarize the findings and logistic regression was carried out to test the associations between the extent of coronary artery disease and the associated factors. There was a firm conclusion that selecting a variable with a cut off p-value of 0.05 as a candidate for multivariate analysis may fail in identifying important variables (10). Besides, it was recommended that a purposive selection of a cutoff value of 0.25 has the capability of retaining both significant covariates and important confounding variables in logistic regression (11). Therefore, this study implemented a purposive selection of all variables with a cutoff value of 0.25 for multivariate analysis. Statistical significance was set at a 5% level.

Ethical approval was obtained from GCMC institutional review board (IRB). The need for informed consent from the study participants was waived by the IRB, owing to the nature of the study. Unique personal identifiers such as the names of the study participants were not revealed maintaining confidentiality of the information collected from the registry.

## Results

This study included a total of 197 patients with coronary artery disease (CAD) that have undergone PCI at GCMC between June 2018 and February 2021. The mean age of the study participants was 58.6 with standard deviation (SD) of 11.5. Of the total number of patients, 159 (80.7%) were males with male to female ratio of 4.2. Dyslipidemia, according to the Third Report of the National Cholesterol Education Program (NCEP), 2002 (12) was documented in 180 patients (91.4%); 114 patients (57.9%) were known diabetic; 51 patients (25.9%) were prediabetic; 109 patients (55.3%) were hypertensive. The mean and SD of study participants' body mass index (BMI) were 26.8±3.3, 152 patients (77.16%) being either overweight or obese. The minority, 29 patients (14.7 %) of the study participants, were smoking. Nearly two-third, 127 patients (64.5%) included in this study were diagnosed to have heart failure (Table 1) and twenty patients (10%) had chronic kidney diseases.

**Table 1:** Baseline characteristics of CAD patients treated with PCI at GCMC, Addis Ababa, Ethiopia

Variables	Characteristics	Frequency (n=197)	Percentage
	Female	38	19.3
**Age in years**	<45	28	14.2
**(Mean=58.6; SD=11.5)**	45–54	40	20.3
	55–64	68	34.5
	≥65	61	31.0
**BMI, kg/m^2^**	<25	45	22.8
**(Mean=26.8; SD=3.3)**	25–29	104	52.8
	≥30	48	24.4
**Smoking**	Yes	29	14.7
	No	168	85.3
**Hypertension**	Yes	109	55.3
	No	88	44.7
**Diabetes mellitus**	Yes	114	57.9
	No	83	42.1
**Prediabetes**	Yes	51	25.9
	No	146	74.1
**Dyslipidemia**	Yes	159	80.7
	No	38	19.3
**CKD**	Yes	20	10.2
	No	177	89.8
**Cardiac status**	Normal	70	35.5
	Heart failure	127	64.5

On presentation, typical chest pain was the predominant presetting complaint in 151 patients (76.6%) followed by fatigue in 145 patients (73.6%), dyspnea and/or orthopnea in 98 patients (49.7%) and atypical chest pain in 38 patients (19.3%) while syncope was a rare presenting complaint occurring in only 4 patients (2%) (Table 2). Aspirin and statins are the most common medications given to our patients before they present to our center, which accounted for 114 patients (58.4%)] and 113 patients (57.4%) respectively followed by beta blockers in 81 patients (41.1%), P_2_Y_12_ inhibitors in 75 patients (38.1%) while angiotensin converting enzyme (ACE) inhibitors are less frequently prescribed, in 51 patients (25.9%).

**Table 2:** Description of presenting complaints among CAD patients treated with PCI at GCMC, Addis Ababa, Ethiopia

Variables	Characteristics	Frequency	Percentage
**Typical chest pain**	Yes	151	76.6
	No	46	23.4
**Atypical chest pain**	Yes	38	19.3
	No	159	80.7
**Dyspnea and/or orthopnea**	Yes	98	49.7
	No	99	50.3
**Palpitation**	Yes	25	12.7
	No	172	87.3
**Fatigue**	Yes	145	73.6
	No	52	26.4
**Syncope**	Yes	4	2.0
	No	193	98.0
**Non-angina pain**	Yes	3	1.5
	No	194	98.5
**Others**	Yes	7	3.6
	No	190	96.4

**Figure 1:**
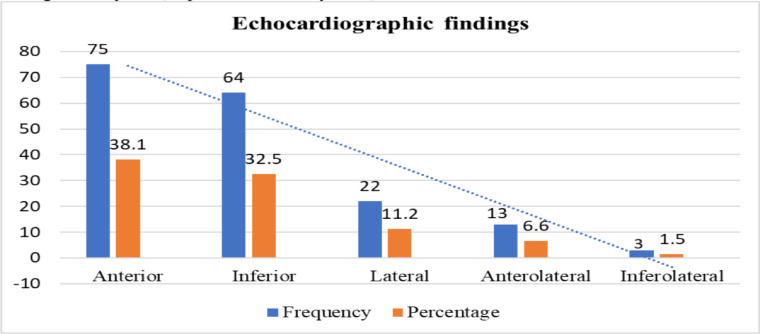
Description of echocardiography findings among CAD patients treated with PCI at GCMC, Addis Ababa, Ethiopia.

The ECG revealed STEMI in 110 of the study subjects (55.8%) followed by ST-T wave changes in 52 patients (26.4%). Completely normal ECG was infrequent, found in only 23 of the study subjects (11.7%) (Table 3). According to the echocardiographic study done before or immediately after the procedure (in primary PCI), the large majority [166(84.3%)] had regional changes as hypokinesis, akinesis or dyskinesis indicating ischemic changes. Anterior wall motion abnormality is the commonest regional change, found in 75 patients (38.1%)] followed by inferior wall motion abnormality [64(32.5%)] (Figure 1).

**Table 3:** Description of ECG findings among CAD patients treated with PCI at GMCC, Addis Ababa, Ethiopia

Variables	Characteristics	Frequency	Percentage
**ECG findings**			
**Normal**	Yes	23	11.7
	No	174	88.3
**STEMI**	Yes	114	57.9
	No	83	42.1
**ST-T wave changes**	Yes	56	28.4
	No	145	73.6
**Left ventricular hypertrophy**	Yes	21	10.7
	No	176	89.3
**Right bundle branch block**	Yes	11	5.6
	No	186	94.4
**Left bundle branch block**	Yes	10	5.1
	No	187	94.9
**Sinus tachycardia**	Yes	6	3.0
	No	191	97.0
**Atrial fibrillation**	Yes	5	2.5
	No	192	97.5
**Significant sinus bradycardia**	Yes	4	2.0
	No	193	98.0
**Others** +	Yes	4	2.0
	No	193	98.0

+ Malignant arrhythmia, supraventricular tachycardia, advanced heart block

**Figure 2:**
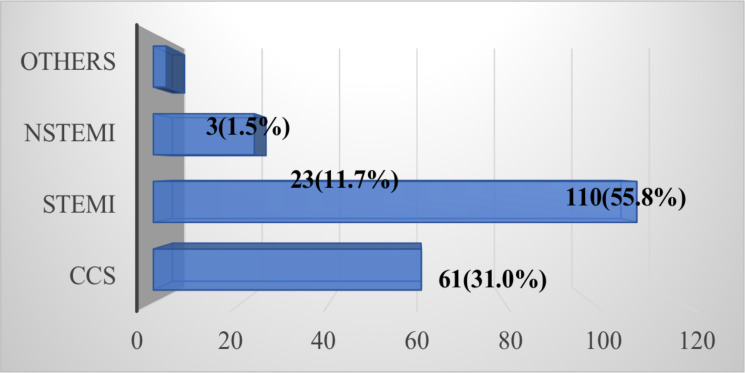
Clinical diagnosis among CAD patients undergoing PCI at GCMC, Addis Ababa, Ethiopia

Figure 2 illustrates that the majority [110 (55.8%)] of our patients undergoing PCI were diagnosed with STEMI followed by CCS [61(31.0%)]. One hundred and twelve patients (56.9%) had emergency/urgent PCI. The majority, [137(69.5%)] of cases, had single vessel intervention while 42 patients (21.3 %), 3 patients (1.5%) and 15 patients (7.6%) had double-vessel, triple-vessel and staged PCI respectively.

On multivariate analysis, statistically significant association was observed between dyslipidemia and the extent of CAD, [AOR 4.1(95%; CI; 1.33–12.48)], implying that patients with dyslipidemia were about four times more likely to be diagnosed with multi-vessel coronary disease compared to their counterparts. The adjusted analysis also showed statistically significant association between patients with left ventricular hypertrophy (LVH) and extent of CAD, [AOR 3.5(95%; CI; 1.22–10.37). Patients with LVH were 3.5 times more likely have multi-vessel coronary disease compared to those without LVH. Age was also associated with extent of CAD on bivariate analysis [COR 3.6 (95%; CI; 1.23–10.68)], the age group of 55–64 years was 3.3 times more likely diagnosed with multi-vessel coronary artery disease compared to those in the age group <45 years. However, age was no more significantly associated after adjustment for confounding variables on multivariate analysis [AOR 2.4(95%; CI; 0.72–7.95)]. Similarly, cardiac status was associated with extent of CAD on unadjusted analysis [COR 2.3(95%; CI; 1.14–4.52)], patients diagnosed with heart failure were 2.3 times more likely to have multi-vessel coronary artery disease compared to those without heart failure on bivariate analysis (Table 4).

**Table 4:** Factors associated with extent of CAD among patients treated with PCI at GMCC, Addis Ababa, Ethiopia

Variables	Extent of CAD		COR (95%CI)	AOR (95%CI)
			
	Multi-vessel	Single vessel		
**Age**				
**<45**	5	23	Ref.	Ref
**45–54**	9	31	1.3(0.40–4.52)	1.04(0.27–3.99)
**55–64**	30	38	3.6(1.23–10.68)*	2.4(0.72–7.95)
**≥65**	16	45	1.6(0.53–5.03)	1.3(0.37–4.56)
**Dyslipidemia**				
**Yes**	55	104	3.5(1.29–9.45)*	4.1(1.33–12.48)*
**No**	5	33	Ref.	Ref.
**Cardiac Status**				
**Normal**	14	56	Ref.	Ref.
**Heart failure**	46	81	2.3(1.14–4.52)*	2.0(0.88–4.48)
**Left ventricular hypertrophy on ECG**				
**Yes**	12	9	3.6(1.41–8.97)**	3.5(1.22–10.37)*
**No**	48	128	Ref	Ref.
**ST-T wave change**				
**Yes**	23	29	2.3(1.19–4.49)*	1.5(0.62–3.58)
**No**	37	108	Ref.	Ref.
**Ischemia on inferolateral region**				
**Yes**	7	5	3.5(1.06–11.47)*	2.1(0.52–8.19)
**No**	53	132	Ref.	Ref.

* p≤0.05

** p≤0.01

In the large majority patients, 169 (85.8%), thrombolysis in myocardial infarction (TIMI) 3 flow grade was achieved while 16 patients (8.1%) had TIMI 2. One hundred and ninety-three patients (98%) were treated without experiencing any complications. Malignant arrhythmia occurred only in one patient. This was an intraprocedure ventricular fibrillation, successfully cardioverted and resolute integrity stent was deployed along the LAD ostial lesion resulting in TIMI 3 flow grade. There was no death within 24 hours of the procedure while a 75-year-old female with STEMI at the proximal LAD and a tight proximal LCX lesion with successful PCI to the former died suddenly on the 3^rd^ post procedure day. There were one ischemic stroke and one major bleeding requiring transfusion following the procedure, both survived without major sequelae. Otherwise, there was no any significant contrast induced nephropathy or contrast reaction. There were 2 deaths documented within 30 days, each of them was death on arrival, postmortem study was not possible.

## Discussion

In this study, the mean age of the study subjects is 58.6 with standard deviation (SD) of 11.5 and a large majority are males, consistent with our previous study in a similar group of study participants (9). Congruent to the aforementioned study and others, there are high frequencies of dyslipidemia, hypertension and diabetes while the other standard risk factors of CVD like smoking and family history of premature CVD are infrequent (5). In our patients, overweight /obesity is also frequent, a consequence of its high prevalence in the urban setting in Ethiopia (13).

Nearly two-third of the study participants had documented heart failure indicating that our patients present late without timely recognition of the grave symptoms of CAD, exposing survivors of the initial events to develop heart failure in the sub-acute and chronic phases. Dyspnea and/or orthopnea is one of the most prevalent symptoms, next to typical angina, further reinforcing our finding that heart failure is the most frequent and alarming clinical condition in CAD patients in Ethiopia. Among patients with AMI, heart failure is most powerful predictor of death and it has important implications for treatment (14).

From the outset, identifying the medications that the patients present with could be an important indicator of the practice pattern in a primary care settings within a particular community. Although aspirin and statins are the most frequent medications documented during their first visits, the frequencies are low, 58.4% and 57.4% respectively, confirming limitations in the application of guideline recommended treatment in our primary care settings, emphasized in a recent review by the lead investigator of this study (15). Most importantly, only 38.1% and 25.9% of our patients were taking some doses of a beta blockers and angiotensin converting enzyme (ACE) inhibitors, respectively, against a high frequency of heart failure, 64.5%.

Consistent with other studies (15,16), normal ECG finding is infrequent among our patients with significant CAD. The majority, 55.8%, of the study subjects had STEMI, in agreement with what was documented in a similar group of patients previously (9). Fifteen percent of AMI patients die from its complications, with STEMI mortality rates superseding NSTEMI rates (5).

Atrial fibrillation is the most frequent sustained cardiac tachyarrhythmia in clinical practice. It is a global health care problem with evidence suggesting an increasing prevalence and incidence worldwide (17,18), infrequent in this study exceeded by RBBB and LBBB which could be a reflection of the occurrence of the different syndromes of CAD at younger ages in Ethiopia. Frequency of RBBB exceeds that of LBBB contrary to the finding in the local study aforementioned above, on the general population of CVD patients presenting to the first private cardiac facility (9). New on-set RBBB in subjects of acute MI is associated with proximal LAD involvement, an ominous condition if timely revascularization is not accomplished (19). In the present study, the higher frequency of RBBB, coexisting with frequent involvement of the anterior wall on echocardiographic study, indicates proximal LAD occlusion leading to STEMI. STEMI equivalents are ECG changes that are highly suggestive of total coronary artery occlusion but lack ST-elevation in contiguous leads (4). One of the conditions that is under investigation for the association of significant occlusion causing MI other than ECG changes from STEMI and STEMI equivalents is RBBB.

In our study, echocardiography revealed left ventricular regional wall motion abnormality as the most frequent change, mostly involving the anterior wall, contributing to the high frequency of left ventricular dysfunction and heart failure. Other studies have demonstrated a higher frequency of heart failure in patients with LAD disease supplying the anterior wall of the left ventricle (20).

Among the various factors suspected to be associated with the extent of CAD, dyslipidemia, LVH and presence of heart failure strongly predicted multi-vessel disease, 4, 3.5 and 2.5 folds respectively. A similar study showed that the various dyslipidemias are significantly correlated with multi-vessel CAD (21). Left ventricular hypertrophy is also documented to predict multi-vessel CAD and heart failure is obviously a mirror image of multiple territory involvement (22).

In most instances PCI was done in the acute or sub-acute phase after ACS, especially in STEMI patients, elevating the frequency of single vessel intervention. In primary PCI, studies have concluded that, failure to restore optimal blood flow in the infarct related coronary artery (i.e., less than thrombolysis in myocardial infarction [TIMI]-3 flow grade) has been noted in 5–23% of patients which has been associated with adverse clinical outcomes (23,24). In the present study, the success rate is enhanced by the fair mix of patients including CCS and non-STEMI ACS cases.

In conclusion, hypertension, dyslipidemia, diabetes and overweight/obesity are the most frequent risk factors in patients with established CAD in Ethiopia while the use of preventive medications in primary care settings is not optimal. Dyslipidemia and LVH are strongly correlated with multi-vessel CAD, alongside a high frequency of post MI heart failure and its predictors underscoring the need for establishment of centers of excellence, besides the implementation of health education programs stressing on primary prevention and timely revascularization. Despite a fairly high frequency of multi-vessel intervention, without surgical back up, the success rate of PCI at Gesund Cardiac and Medical Center is praiseworthy
